# RNA Targeting in Inherited Neuromuscular Disorders: Novel Therapeutic Strategies to Counteract Mis-Splicing

**DOI:** 10.3390/cells10112850

**Published:** 2021-10-22

**Authors:** Veronica Verdile, Gloria Guizzo, Gabriele Ferrante, Maria Paola Paronetto

**Affiliations:** 1 Laboratory of Molecular and Cellular Neurobiology, Fondazione Santa Lucia, CERC, 00143 Rome, Italy; v.verdile@hsantalucia.it (V.V.); gloriaguizzo95@gmail.com (G.G.); gabry87.gf@gmail.com (G.F.); 2 Department of Movement, Human and Health Sciences, University of Rome Foro Italico, Piazza Lauro de Bosis 6, 00135 Rome, Italy

**Keywords:** alternative splicing, neuromuscular disease, RNA-based therapies

## Abstract

Neuromuscular disorders represent multifaceted abnormal conditions, with little or no cure, leading to patient deaths from complete muscle wasting and atrophy. Despite strong efforts in the past decades, development of effective treatments is still urgently needed. Advent of next-generation sequencing technologies has allowed identification of novel genes and mutations associated with neuromuscular pathologies, highlighting splicing defects as essential players. Deciphering the significance and relative contributions of defective RNA metabolism will be instrumental to address and counteract these malignancies. We review here recent progress on the role played by alternative splicing in ensuring functional neuromuscular junctions (NMJs), and its involvement in the pathogenesis of NMJ-related neuromuscular disorders, with particular emphasis on congenital myasthenic syndromes and muscular dystrophies. We will also discuss novel strategies based on oligonucleotides designed to bind their cognate sequences in the RNA or targeting intermediary of mRNA metabolism. These efforts resulted in several chemical classes of RNA molecules that have recently proven to be clinically effective, more potent and better tolerated than previous strategies.

## 1. Introduction

Pediatric neuromuscular disorders are rare heterogeneous inherited diseases characterized by abnormal muscle function due to alterations or lesions arising from the motor unit, which is composed by innervated muscle fibers, neuromuscular junction (NMJ) and motor neurons. Neuromuscular disorders represent multifaceted abnormal conditions, with little or no cure, leading to patient deaths from complete muscle wasting and disabilities [1,2]. They are classified as forms affecting the muscle itself (myopathies and dystrophies), forms affecting the NMJ (myasthenia) and forms affecting the motor neurons (spinal muscular atrophy (SMA) and Charcot-Marie Tooth [1,2,3]). 

**Figure 1:**
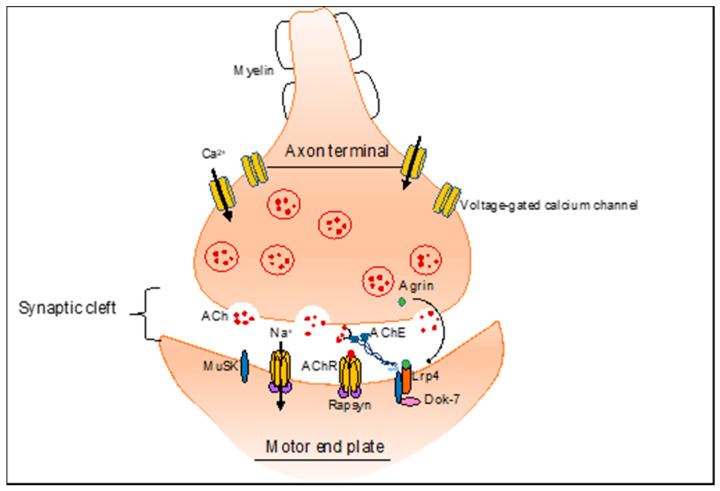
Schematic representation of the neuromuscular junction. Nerve impulse transmission is ensured by the voltage-gated calcium channel, AChR and AChE. The increase in Ca^2+^ facilitates the fusion of synaptic vesicles with the presynaptic membrane and the consequent release of ACh in the synaptic cleft. The ACh-AChR complex induces opening of the ligand-gated ion channels and muscle membrane depolarization, thus generating muscle contraction. Signal transmission is interrupted by the breakdown of ACh, catalyzed by the AChE, which is anchored to the postsynaptic membrane through MuSK. Furthermore, MuSK forms a complex with Lrp4 and Dok-7, which binds the neural agrin and induces a phosphorylation cascade. Phosphorylated MuSK triggers phosphorylation of other intracellular proteins, such as rapsyn, thus promoting the formation of the AChR cluster, which is necessary for the maintenance of the postsynaptic structure.

Proper formation and functionality of the NMJ represent the basis for a successful neuromuscular transmission. NMJ is the highly specialized synapse between a nerve terminal of the motor neuron and its innervated muscle fibers, responsible for converting the electrical impulses generated by the motor neuron into electrical activity in the muscle fiber [1]. Like other chemical synapses, NMJs are composed by proteins important for nerve impulse transmission and proteins involved in the functionality of the postsynaptic membrane. The transmission is ensured by the acetylcholine receptor (AChR) subunits, acetylcholinesterase (AChE) and voltage-gated sodium channels, that convert electrical impulses into action potentials, propagated along the muscle fiber [4]. NMJ development and maintenance are guaranteed by *core* NMJ proteins, like agrin, muscle-specific kinase (MuSK), low-density lipoprotein receptor-related protein 4 (Lrp4), downstream of tyrosine kinases-7 (Dok-7), and 43 kDa receptor-associated protein of the synapse (rapsyn), in addition to auxiliary proteins that support NMJ correct functionality, including neuregulins, AChR and components of the dystrophin-glycoprotein complex (DGC) [1] (Figure 1).Defects in these molecules can lead to impaired neuromuscular transmission, driving the onset and progression of neuromuscular pathologies, including myasthenia and muscular dystrophies [2,5,6,7].

Remarkable progress in the past decades defined causative mutations driving the pathogenesis of inherited neuromuscular diseases, supported by mechanistic studies unveiling the consequences of these mutations [2]. However, targeting of the molecules involved with therapeutic purpose led to poor clinical responses. Current strategies are also attempting to pharmacologically block the consequences of the dystrophic process, including the prevention of the oxidative stress or inhibition of the chronic activation of the NF-κB signaling concomitant with muscle fiber breakdown and inflammation [8]. Corticosteroids have shown to improve dystrophic muscle strength and function in infants, young children, and adults [9]. Unfortunately, long-term daily corticosteroid use also brings with it multiple side effects including weight gain, osteopenia Cushingoid features, and delayed puberty [9]. Overall, a coordinated, multidisciplinary approach seems essential for optimum management of these diseases.

To further address this challenge, recent therapeutic efforts have focused on RNA as the mediator of pathogenesis, using state of art technologies to drug aberrant transcripts. 

We review here recent progress on the role played by alternative splicing (AS) in ensuring functional NMJs, and its involvement in the pathogenesis of NMJ-related neuromuscular disorders, with particular emphasis on congenital myasthenic syndromes and muscular dystrophies.

## 2. NMJ-Related Neuromuscular Disorders

NMJs are cholinergic synapse where the release of acetylcholine from motor nerve terminals generates a local endplate potential on the muscle fiber, that depolarize the entire muscle fiber and initiate the process of excitation-contraction coupling [10]. Deficits of neuromuscular transmission can result in muscle weakness and atrophy, due to alterations in proteins essential for muscle integrity and/or function [11,12,13,14]. For instance, the loss of dystroglycan in DGC prevents agrin binding and disorganizes AChR clustering [1,13]. Moreover, reduced MuSK expression or its partial inactivation accelerates the loss of AChRs and impairs neurotransmission [15,16]. Typical pathogenic conditions with impaired NMJ are the Congenital Myasthenic syndromes (CMS) and Muscular Dystrophies (MD) [1].

Congenital Myasthenic syndromes represent a large group of genetic disorders characterized by muscle fatigue caused by communication defects between nerves and muscle fiber [17]. To date, pathogenic variants of 32 genes are known to cause autosomal dominant or recessive CMSs [18]. All subtypes of CMS share the clinical features of fatigability and muscle weakness, but age of onset, symptoms, and response to treatment vary according to the specific genetic mutation [18]. The most frequently mutated gene in CMS is represented by *CHRNE*, coding for the ε subunit of nicotinergic post-synaptic receptor AChR, resulting in primary AChR deficiency. A reduced number of functional AChRs on the post-synaptic membrane is also caused by mutations in the *CHRNA1* gene, coding for the α subunit of the AChR, leading to an imbalance between the two splice variants (P3A− and P3A+) produced [18]. More rarely, mutations occur in the *CHRN1B, CHRND, CHRNG* genes, coding for the β-/δ-/and fetal γ-subunit, respectively. AChR, which is expressed on the crests of the folds of the postsynaptic membrane, binds ACh, a neurotransmitter contained in the synaptic vesicles and released from the motor nerve terminal, which merge with the membrane at specialized sites called active zones. Frequently, mutations occur in *CHAT, COLQ, RAPSN, DOK7* and *GFPT1* genes, thus affecting transmission [18]. 

Muscular dystrophies (MD) represent a wide range of specific myopathies, typified by the pathological presence of the dystrophic muscle, characterized by progressive tissue weakness and degeneration [19,20]. When the degeneration exceeds the ability of muscle regeneration, muscle atrophy occurs, and muscle tissue can be replaced by connective and adipose tissue [21,22]. Although MD patients show similar clinical characteristics, at present more than 50 different forms of MDs are recognized, classified for the specific gene mutations, different affected muscles, and/or involvement of other organs [23]. Among them, the most frequent forms are represented by dystrophinopathies (Duchenne Muscular Dystrophy (DMD) and Becker Muscular Dystrophy (BMD) and myotonic dystrophy (DM)) [23].

Dystrophinopathies are X-linked recessive diseases, affecting 1 in 5000 to 1 in 6000 live-born males. Except for the extremely rare event of recessive homozygosity, females are asymptomatic carriers, because the healthy X chromosome can offset the faulty one and only occasionally can they experience milder symptoms [19,24]. Both DMD and BMD are caused by mutations in the *DMD* gene, encoding for dystrophin, a protein that connects cytoskeletal F-actin of the muscle fiber to the surrounding extracellular matrix [25]. 

DMD is an extremely severe pathology with symptom onset in early childhood, while BMD manifests in a milder form, with a late onset and slower progression compared to DMD [19]. This phenomenon, explained by the reading frame rule, correlates with the type of mutations. Indeed, DMD shows out-of-frame mutations or nonsense mutations, producing unstable or truncated dystrophin proteins, whereas BMD mutations maintain the reading frame and produce a partially functional dystrophin [19]. However, exceptions of the reading frame rule have been observed in 10% of patients, displaying point and nonsense mutations [26,27].

Genetic neuromuscular disorders include also myotonic dystrophy (DM), an autosomal dominant disease manifesting in two genetic types based on the location of the mutation: DM1 and DM2 [20]. DM1, also known as Steinert’s disease, is caused by a CTG triplet repeat expansion in the last exon of *DMPK* gene, encoding the myotonin-protein kinase (MT-PK), which plays an important role in regulating the production and function of muscle structures by interacting with other proteins and in the communication of muscle, heart and brain cells [20]. Healthy individuals carry less than 37 triplet repeats, whereas longer expansions become unstable and tend to grow somatically and intergenerationally. This underlies the genetic anticipation phenotype, which leads to early onset of symptoms and more severe disease [20]. This is the case of congenital myotonic dystrophy (cDM), due to the extreme triplet repeat expansion inherited from an affected mother, which represents the most dramatic manifestation of the pathology [23]. 

Expanded-CUG-transcripts are retained in the cell nucleus and accumulate as focal aggregates, sequestering splicing regulators, including CUG-binding protein 1 (CUGBP1) and muscleblind-like 1 (MBNL1). These proteins are involved in the processing and localization of RNA, particularly for contractile protein synthesis. Furthermore, this accumulation of RNA alters the splicing process, as it also sequesters cleavage and splicing factors, such as hnRNPs and snRNPs, strengthening the hypothesis that the dystrophic phenotype of patients with DM1 may result from a more general alteration of the pre-mRNA post-transcriptional processing [28].

DM2 is characterized by CCTG expansion repeat in the first intron of the Cellular Nucleic acid Binding Protein/Zinc Finger Protein 9 (*CNBP/ZNF9*) gene, on chromosome 3q21. Unlike DM1, DM2 has no congenital onset. It exhibits a mild phenotype, often not diagnosed or diagnosed late, when patients have reached adulthood [20]. 

Epidemiology and symptoms of the mentioned neuromuscular disorders are summarized in Table 1.

## 3. Alternative Splicing in the Skeletal Muscle

Alternative pre-mRNA splicing is one of the main mechanisms of gene expression regulation, which expands the genomic coding capacity and the proteomic diversity in eukaryotic cells [29,30]. Most human genes undergo AS, thus explaining, at least in part, the discrepancy between the relatively small number of genes and the complexity of the human proteome [31,32]. Alternative spliced transcripts give rise to proteins often displaying differences in subcellular localization, post-translational modifications and/or functions [33,34,35,36,37]. During development, AS contributes to organ maturation by producing temporal-specific protein isoforms. In addition to the spliceosome, which is an enzymatic ribonucleoprotein complex, AS is finely regulated by many RNA-binding proteins (RBPs) [38]. RBPs help the spliceosome during splicing reactions, thus promoting the inclusion or exclusion of different exons in the mature transcripts, depending on the specific cell- and tissue- context. Interestingly, several of these factors are precisely regulated in specific tissues and during developmental stages in terms of expression levels, localization, their own splicing, mRNA stability and translation efficiency [29,39]. AS represents a ubiquitous regulatory mechanism of gene expression, which contributes to cell differentiation and lineage determination, acquisition and maintenance of tissue-identity, and organ development [40]. Numerous studies agree in reporting that skeletal muscle, brain and heart show the highest levels of tissue-specific AS, which is also highly conserved during evolution [40,41,42]. Indeed, physiological changes that occurs during skeletal muscle development are accompanied by numerous transcriptional and post-transcriptional modifications, including AS modulation [43,44]. 

In recent years, global transcriptome profiling highlighted extensive transcriptional- and post-transcriptional changes during myogenesis, including splicing alterations [45,46,47]. In particular, it was shown the important role played by AS in sarcomere assembly, stability, and contractility. Sarcomere components, like tropomyosin (TM), troponin (TnT), Titin and Myosin binding protein-C (MyBP-C) are highly affected by AS mechanism [48]. For instance, TM is expressed in all cell types, but AS generates tissue-specific isoforms [49]. Indeed, the mutually exclusive inclusion of exon 5 in the *α-TM* gene generates either the α-TM SK or α-TM NM variants. The first variant is restricted to skeletal muscles; on the contrary, the other is highly expressed in non-muscle cells [50,51]. 

Functionally, the skeletal muscle specific isoforms are important for modulating the kinetics of muscle contraction within muscle fibers [52]. In this context, a pivotal role is played by the membrane components and tubular invaginations. Indeed, membrane trafficking proteins regulate distinct processes, such as internalization, recycling and degradation of ion channels, receptors, and membrane components. Several genes encoding membrane trafficking and endocytosis proteins are alternatively spliced during mouse skeletal muscle development [53]. 

Thus, alternative splicing allows and guarantees functional diversity and specific properties of muscle-generating capacity. 

## 4. Alternative Splicing and Neuromuscular Genetic Diseases 

Splicing abnormalities and defects in the spliceosome machinery or in the RBPs are known to influence the pathogenesis of human diseases and can represent a key factor driving disease susceptibility and severity. Given the relevance of the splicing process in promoting specific function and correct development of the skeletal muscle, defects in splicing mechanism and in the RBPs are known to influence the pathogenesis of muscular and neuromuscular diseases. Aberrant AS can contribute to the pathogenesis of human diseases through different mechanisms: mutations of the core splicing consensus sequences in the intron-exon boundaries (thus preventing the binding of trans-acting factors), alterations of the expression of RBPs and/or their sequestration [54].

### 4.1. Alternative Splicing Defects in Congenital Myasthenic Syndrome (CMS)

As described above, CMSs are a group of NMJ disorders compromising the neuromuscular signal transmission. Aberrant splicing deeply contributes to the pathogenesis of CMS, affecting physiological integrity and function of the NMJ [55]. The majority of the AS defects in CMS arise from mutations at the consensus splicing cis-element, which determine a lower binding affinity for trans-acting factor, compromising the recognition of a splice site [56]. For instance, several splicing mutations fall into genes encoding subunits of the acetylcholine receptor (AChR). Most of the mutations occur in the *CHRNE* gene, affecting the 5′ and 3′ splice site of different introns and leading to intron retention or exon skipping events in the mature transcript [57]. 

Several mutations associated with CMS disrupt the auxiliary splicing cis-elements (ISEs/ESEs and ISSs/ESSs). These mutations can silence, enhance, or change the activity of splicing regulatory factors [57]. For example, the mutation ɛEF157V in *CHRNE* exon 6 causes skipping of exon 6 itself, maybe altering the binding of specific RBP [57]. Another single nucleotide mutation able to disrupt binding site for specific RBP has been identified in *ColQ* gene, which encodes for collagen-like tail subunit (collagen Q). This is essential for the anchoring of acetyl cholinesterase (AChE) to the basal lamina of the NMJ. In particular, the A-to-G transition in *ColQ* exon 16 disrupts the binding of the splicing-enhancing SRSF1, and *de novo* gains binding of the splicing-suppressing hnRNP H, thus leading to skipping of constitutive exon 16 [56].

Several mutations linked to CMS can also alter splicing process by generating a *de novo* splice site and activating a cryptic splice site. For example, c.513C>T in exon 4 of *DOK7* generates a GT dinucleotide within exon 4, which is used as a novel splice donor site [58]. Similarly, IVS1-15C>A in *RAPSN* gene *de novo* generates an AG dinucleotide in intron 1 and causes retention of 13 nucleotides in intron 1 [59]. In exon 6 of *CHRNE* pre-mRNA the ɛE154X mutation leads to the activation of a cryptic 3′ splice site, and to the loss of ESE [57]. Moreover, the IVS6-1G>C mutation in intron 6 of *CHRNE* inactivates the native 3′ splice site and abnormally activates a cryptic 3′ splice site in exon 7 [57].

Other interesting splicing mutations in CMS are relevant to cryptic exonization, in which a transposable element (TE) and mammalian interspersed repeats (MIRs) are changed to an exon during evolution [60]. An example is exon P3A in *CHRNA1* gene, encoding the AChR α1 subunit. Exon P3A and its flanking intronic regions originate from exonization of the retroposed mammalian interspersed repeat element (MIR), which was inserted between exons 3 and 4 of *CHRNA1* [60]. The inclusion of exon P3A generates a non-functional AChR α1 subunits, that cannot be incorporated into AChR, thus impeding the cell surface expression of AChR. Notably, preferential inclusion of exon P3A was confirmed in the patient’s muscle together with the significant decrease in the density and distribution of the AChR [61]. Mutations in this exon alter the binding affinity of regulatory splicing factors hnRNP H, PTBP1 and hnRNP L, which compete as antagonist binding trans-factors because of the presence of overlapping motifs [61]. 

On the contrary, hnRNP C, YB-1, and hnRNP L cooperate to enhance skipping of exon 10 in *MuSK* transcript [62]. MuSK mediates AChR clustering at the motor endplate and exon 10 encodes a frizzled-like cysteine-rich domain (Fz-CRD), which is essential for Wnt-mediated AChR clustering. Mutations in cis-elements of exon 10 potentially affect the function of MuSK by changing AS of exon 10, and can consequently inhibit neuromuscular signal transmission [62]. 

Collectively these reports highlight the important role of splicing for NMJ formation, maintenance and function. 

### 4.2. Alternative Splicing Defects in Duchenne Muscular Dystrophy (DMD)

DMD is the most common and severe form of all muscular dystrophies, caused by mutations in the DMD gene, which prevents the synthesis of the dystrophin [25]. The absence of dystrophin compromises the integrity of the sarcolemma, making it more susceptible to injury during muscle contractions or stretch, and leading to muscle degeneration with impaired regeneration [25]. Approximately 60–70% of DMD patients have deletion mutations in one or more exons [63]. This could be explained by the feature of *DMD locus*. Indeed, *DMD* is the longest known gene in the human genome with 2.4 Mb [64]. However, the mature mRNA of dystrophin is only 14 kb long, due to the presence of short 79 constitutive exons flanked by long introns [65]. Disease mutations are spread across the 79 *DMD* exons, although exon 45–55 represent specific ‘hotspot’ regions where deletions are particularly frequent (70% of deletions fall in this region) [66]. Additionally, *DMD* exons 45–55 deletion is known to be associated with a remarkably mild phenotype [66], called Becker muscular dystrophy (BMD). Indeed, this deletion is an in-frame mutation, leading to the production of shorter and partially functional dystrophin isoforms [65,67,68]. Moreover, genomic deletion of a single exon (44) or multiple exons (44–45) in *DMD* gene generates out-of-frame mutations [69]. The novel sequence created at the junction between introns 44 and 45 after removal of exon 45 promotes the recruitment of the hnRNPs, which determine the skipping of exon 44, thus restoring an open reading frame. Partial restoration of an in-frame transcript arises also by exclusion of exon 45 in transcripts lacking exon 44 [68]. This is due to the lack of splicing factors CELF2a, required for proper exon 45 recognition in the splicing process [68]. In addition, mitigation of the pathological phenotype can occur through skipping of the nonsense mutation-containing exons. This allows the rescue of the correct open reading frame in the dystrophin transcripts. For example, high level of nonsense-induced exon skipping around exons 25–40 generally results in a mild DMD phenotype, and some cases in an almost asymptomatic phenotype [70]. The molecular mechanisms that enable exon skipping are multiple. Indeed, mutation can induce changes in cis-regulatory elements in term of density and distribution, and/or in strength of signals in the mutated exon. Hence, these changes alter the recognition of exon in the mature RNA, leading to its total or partial exclusion from the mature transcripts [26,71].

Several studies reported that most of intronic mutations in the *DMD* gene create a novel splice site and/or increase the strength of an existing sub-optimal splice-site, promoting the introduction of cryptic exons into the mature transcript [72]. Interestingly, it has been demonstrated that about half of the cryptic exons associated with disease, derive from transposable elements, particularly Alu elements, which rearrange into the gene by accumulating mutations that generate splice sites [72,73]. For instance, Alu-like mobile elements rearrange into the dystrophin gene and activate one cryptic splice site in intron 11. The alternative transcript produced reveals a truncated translation due to the numerous stop codons present in every frame of the Alu-like sequence [74]. Another example of cryptic exon generated by intronic mutation regards the intron 44 of *DMD* gene. Intron 44 represents a major recombination and deletion hot spot in the *DMD* gene, providing several examples of abnormal splicing events [75]. In fact, the in-frame genomic deletion of exons 45–47, leads to the out-of-frame insertion of a cryptic exon, derived from intron 44, determining a severe phenotype in patients [76]. The junction of introns 44 and 47 lies within the splice donor site of cryptic exons and reinforces its strength. On the contrary, silencer elements, normally located downstream the donor splice site of the cryptic exon, are lost [76]. Similarly, the junction sequences of an in-frame genomic deletion of exons 35–42 arrange splice sites in a suitable configuration favoring the recognition of an intronic sequence as a novel exon. This results in its abnormal inclusion in the mature transcript [75].

Collectively, these findings suggest that manipulating the splicing of dystrophin pre-mRNA to induce the skipping of specific exons can restore a correct reading frame, representing an exploitable strategy for DMD treatment.

### 4.3. Alternative Splicing Defects in Myotonic Dystrophy Type 1 (DM1)

Several studies have highlighted the strong impact of splicing defects on the DM1 pathogenesis, contributing to myotonia, muscle weakness, muscle wasting, insulin resistance, and defective calcium ion channels. As described above, DM1 is caused by expanded CUG repeats in the DM protein kinase (DMPK1) gene transcripts [77]. These expanded microsatellite repeats lead to the formation of a stable RNA secondary structure that sequesters the splicing regulator MBNL, leading to misregulated splicing pattern in several mRNAs [77,78,79,80,81]. For instance, sequestration of MBNL proteins causes skipping of the alternative exon 11 in *INSR* pre-mRNA, thus promoting the switch from the isoform *INSR-B*, which includes the exon 11 and is expressed in the skeletal muscle, into the isoform *INSR-A*, usually expressed in brain, spleen and leukocytes [82]. The inappropriate expression of *INSR-A* in skeletal muscle, which encode for lower-response insulin receptor [83], correlates with the insulin resistance in DM1 patients [84]. Loss of MBNL function in DM1 skeletal muscle also alters the splicing of *CLCN1* gene, leading to myotonia. This is due to the in-frame inclusion of intron 2 and exon 7a in the mature mRNA, leading to the formation of a premature stop codon. The truncated protein is unable to locate in the surface membrane, producing a chloride channelopathy that determine membrane hyperexcitability [77]. In addition to these aberrant splicing events, *de novo* sequestration of MBNL proteins causes the skipping of penultimate exon 78 of the *DMD* pre-mRNA. Loss of exon 78 encodes for an embryonic isoform of dystrophin that causes abnormally ringed muscle fibers with disorganized Z band [85]. This isoform promotes instability of the sarcolemma and may contribute to muscle weakness. 

The sequestration of MBNL promotes splicing alteration in bridging integrator-1 (*BIN1*) transcript. Indeed, both experiment of MBNL1 depletion by an siRNA-mediated approach and overexpression of ~1000 CUG or 300 CCUG repeats shown the skipping of exon 11, generating the embryonic and inactive isoform. This isoform leads to disorganized T-tubule structures, thus impairing the excitation–contraction coupling the sarcomere, ultimately leading to muscle weakness [77].

Sarcolemma stability is ensured by the dystrophin-glycoprotein complex (DGC), and several studies reported that mis-splicing of components of DGC can lead to muscular dystrophy [13,86]. For instance, dystrobrevin (DB) exists as α-DB and β-DB isoform. α-DB undergoes AS to generate five different isoforms, among which α-DB1 and α-DB2 are the most represented and are expressed exclusively in muscle cells [87]. However, these two isoforms exhibit different C-terminus and diverse localization. In fact, α-DB1, which contains exons 1 to 21, is mainly located at the synapse, while α-DB2, which contains exons 1 to 17b and lacks COOH-terminal domain, is localized to sarcolemma of the NMJ. Both isoforms are indispensable for muscle signaling and neuromuscular synaptogenesis. In DM1 patients, splicing of α-DB1 is dysregulated, showing an enhanced inclusion of exons 11A and 12, encoding the variable region 3 [88]. This event results in altered phosphor-tyrosine signaling at the NMJ [88].

Concerning the sarcomere excitation–contraction (EC) coupling elements, it has been demonstrated that aberrant splicing occurs in genes involved in the maintenance of the intracellular calcium homeostasis, playing a key role in muscle degeneration in DM1 [89,90,91]. The calcium channel CaV1.1, coded by the *CACNA1S* gene, is a voltage-sensitive channel that plays a central role in excitation–contraction coupling. Skipping of exon 29 generates an increased CaV1.1 conductance and voltage sensitivity, enhances calcium influx and leads to a spontaneous calcium sparklets in muscle fiber during EC coupling. This entails a reduced force and enhanced endurance, as observed in myotonic dystrophies DM1 and DM2 [89,91,92]. Another protein involved in calcium homeostasis with reported splicing alterations in DM1 is the RYR1. During the normal skeletal muscle contraction/relaxation cycle, calcium is released from the sarcoplasmic reticulum into the cytoplasm through RYR1, inducing muscle contraction. The fetal RYR1 (RyR1-ASI(−)), is increased in DM1 tissues compared to control samples [90]. RyR1 ASI(−) displays lower activity, diminishes the stably opening of channel, thus leading to a significantly lower incidence of Ca^2+^ oscillations, which in turn determines reduction of muscle contraction [90]. DM1 patients show also aberrant AS in the *SERCA1* transcript (*ATP2A1*) [90]. Like for *RYR1*, the neonatal form *SERCA1b*, which lacks exon 22, is exclusively expressed in fast twitch fibers in DM1 pathogenesis. The adult isoform, *SERCA1a* (which includes exon22), was found only in non-DM muscles. Moreover, *SERCA2d*, which retains intron 19, is down-regulated in DM1 muscle tissue [90]. These events contribute to the alteration of the intracellular calcium homeostasis and muscle degeneration in DM1 [90]. 

## 5. RNA-Based Approaches to Address Splicing Alterations

In the last 20 years, remarkable steps forward have been made in the study of inherited neuromuscular diseases, revealing that most identified genes are not readily targeted with current small-molecule therapeutics, thus pushing for new strategies. In this context, RNA represents a unique target for developing therapeutics, in terms of applicability and efficiency of the drug-discovery process. Rather than replacing current therapies, RNA-based antisense technologies aim at complementing small molecules and protein-based drugs by targeting RNA instead of proteins. RNA targeting greatly expands the number and classes of targets that can be approached [93].

As described above, point mutations in genes encoding RBPs and alterations in their expression levels can cause splicing aberrancies, which in turn contribute to the outcome of the disease. Several RNA-based strategies able to revert splicing defects to a physiological state have been recently developed [93,94], including RNA interference (RNAi) [95], antisense oligonucleotides (ASOs) [95], genome editing, achieved through CRISPR-based therapeutics [96], and miRNA-based strategies [97].

Among the new RNA-based therapeutic strategies, ASOs are the most promising molecules, and they were the first oligonucleotide-based therapies achieved for DMD (Eteplirsen and Golodirsen, see above) [98] and SMA (Nusinersen) [93,99].

Two biochemical classes of ASOs are commonly used today: (1) single-stranded ASO targeting RNA through the enzyme RNase H or modulating splicing intermediates [100], and double-stranded synthetic oligonucleotides that work through the RNA-induced silencing complex (RISC) to promote degradation of the target RNA [100]. Once bound to the target RNA, ASOs trigger degradation via the recruitment of endogenous RNase H1, or, in the case of siRNA, the RISC complex. Several chemical modifications have been introduced to improve stability and delivery efficacy, including phosphorothioate with one or more 2′-ribose sugar modifications (2′-MOE, cEt, LNA, and 2′-OMe) or with sugar-phosphate modifications (e.g., morpholino and PNA) [100,101].

In this last part of the manuscript, we describe state of art approaches to address splicing alterations in neuromuscular disorders (Table 2). However, despite the improvements in ASO chemistry and design, systemic use of ASOs is still limited because uptake in many tissues, including skeletal and cardiac muscles, is not sufficient for proper targeting [93]. 

### 5.1. RNA-Based Approaches in CMS

Mutations disrupting cis-acting splicing elements or compromising catalytic functions of trans-acting RBPs impair splicing efficiency, thus causing pathological consequences. Splicing is a druggable process, exploitable for therapeutic purpose. In Congenital Myasthenic Syndrome (CMS), the diagnosis of different subtypes driven by different mutations has rendered quite arduous the development of ASO strategies. Several patients show aberrant AS of *CHRNA1* gene, driven by intronic or exonic point mutations, leading to an increase in P3A(+) and a concomitant decrease in P3A(−). ASOs complementary to these intronic and exonic sequences spanning the exon P3A have been developed and tested with recombinant minigenes [102]. Notably, ASOs covering the 5′ splice site resulted most promising in the rescue of P3A skipping [102], probably through competition with U1snRNP recruitment. The same result of P3A skipping was obtained with the endogenous *CHRNA1* gene upon transfection of the 5′ splice site ASO, confirming its efficacy [102].

### 5.2. RNA-Based Approaches in DMD

**Figure 2:**
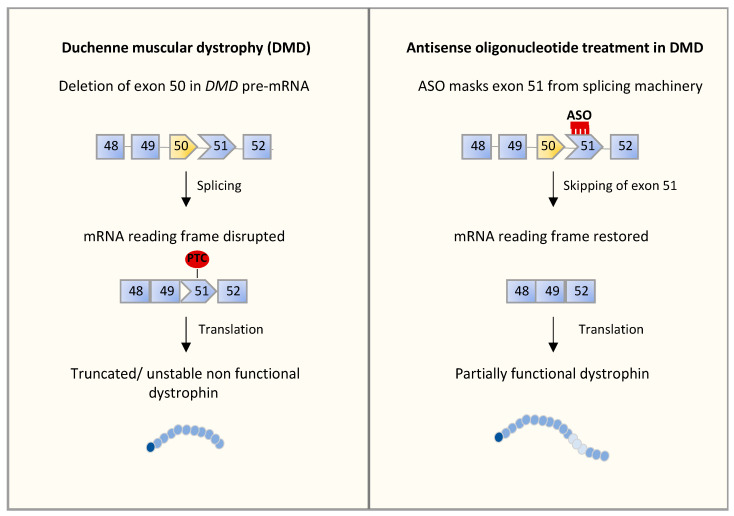
Antisense-oligonucleotide treatment for Duchenne muscular dystrophy (DMD). Patients with DMD display mutations which disrupt the open-reading frame of the dystrophin pre-mRNA. Schematic representation of DMD pre-mRNA from exon 48 to 52 is shown. Genomic deletion of exon 50 leads to an out-of-frame mRNA generating a premature stop codon. This results in the synthesis of a truncated non-functional dystrophin (**left** panel). Eteplirsen (ASO) specifically recognizes sequences of exon 51 of the *DMD* gene, allowing its exclusion from the mature mRNA. This restores the open-reading frame, promoting the synthesis of an internally deleted but partially functional dystrophin (**right** panel).

Several efforts have been directed to promote skipping of specific dystrophin exons and restoration of dystrophin functionality by using the ASO technology [103]. Since several exons can be affected in DMD, no single oligonucleotide can address all forms of the disease [93,101]. ASOs designed to promote skipping of exon 51 are the most advanced in clinical trials. In patients displaying out-of-frame deletions or duplications, the reading frame can be restored by removal of an adjacent exon, thus generating an in-frame, although internally deleted, protein. To achieve this goal, two ASOs have been developed intended to do the same thing, but differed in their chemical structures (Figure 2): the Eteplirsen (EXONDYS 51^®^), which is a phosphorodiamidate morpholino oligomer antisense oligonucleotide designed to bind to exon 51 [98], and Drisapersen (Kyndrisa), a uniform 2′-O-methyl PS-modified oligonucleotide designed to bind to a sequence within exon 51, both promoting skipping of the exon [104]. Notably, preliminary results of a phase 3 post-approval trial of Eteplirsen demonstrated increased dystrophin expression. Both drugs were submitted to the US FDA for market authorization. Eteplirsen received accelerated approval [105], whereas Drisapersen was rejected on the basis of toxicity [106]. In fact, although the initial results were encouraging, with Duchenne boys improving their walking ability, as the study continued, the difference between treated and control patients became smaller. Moreover, reactions of skin fibrosis or fragility and negative impact on kidneys were registered, leading to rejection from the FDA [106].

In addition to exon 51, other potential targets for exon skipping in DMD are exons 45 and 53. Casimersen is a phosphorodiamidate morpholino oligomer that targets exon 45 [107], and received its first approval in the USA by FDA on 25 February 2021 [108]. It was intended for treatment of DMD patients having exclusively a mutation derived from exon 45 skipping [70]. Viltolarsen (VILTEPSO^®^) and Golodirsen (VYONDYS 53™), conversely, target exon 53 [109]. Golodirsen was approved in the USA by the FDA in December 2019 based on positive results from a phase I/II clinical trial [110], while Viltolarsen received a conditional approval on August 2020 from the FDA and recruited for phase II-III clinical trials [110,111]. Exon skipping has also been achieved through viral delivery of U7 small nuclear RNAs, displaying a more efficient and durable effect [112]. U7 is a non spliceosomal snRNA normally involved in the processing of the histone mRNA 3′ end; it can be engineered to bind the appropriate spliceosomal Sm proteins and redirect splicing choices [113]. Notably, a phase 1 trial of U7 small nuclear RNA therapy in patients with DMD was launched in January 2020, providing a single dose of scAAV9.U7.ACCA small RNA systemically delivered via a peripheral vein injection (NCT04240314) [114].

### 5.3. RNA-Based Approaches in DM1

Several RNA-based strategies have been engineered to target CUG expanded RNA transcripts in DM1. As mentioned, in DM1, transcripts from the mutant allele contain expanded CUG repeats, which cause its retention in the nucleus along with splicing factors of the MBNL family, thus exerting a toxic gain-of-function [77]. MBNL sequestration leads, in turn, to AS misregulation [115]. 

#### 5.3.1. Antisense Oligonucleotides in DM1

**Figure 3:**
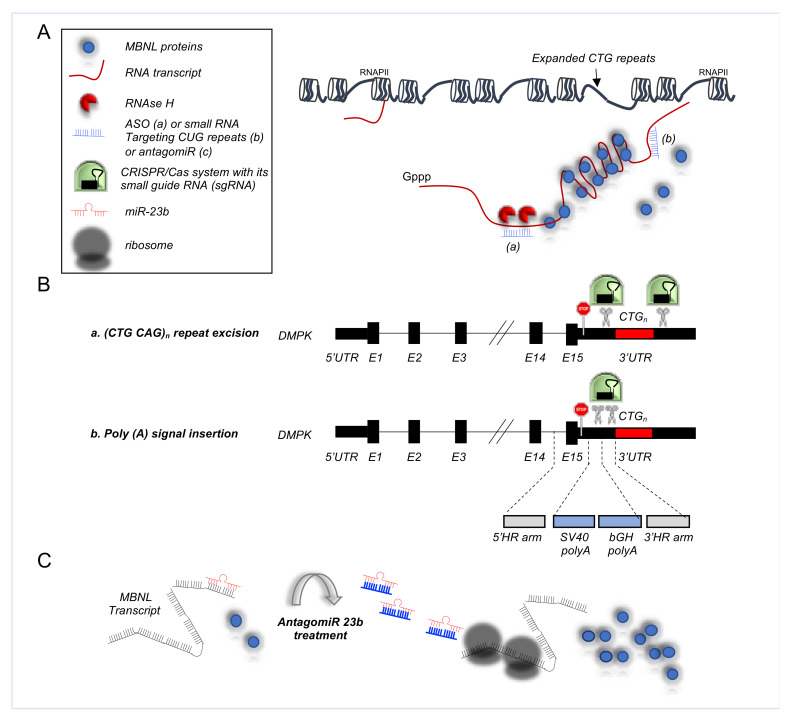
RNA based approaches in DM1. (**A**). ASO strategies in DM1: Once bound to the target RNA, ASOs trigger degradation via the recruitment of endogenous RNase H1 (**a**) or the RISC complex. On the other hand, ASOs complementary to CUG repeats can block MBNL recruitment (**b**) (adjusted from [116]). (**B**). 2 CRISPR/Cas9 based therapies for DM1. CRISPR/Cas system interferes with the genome via a small guide RNA (sgRNA), directing the Cas endonuclease (in green) to a DNA target that matches the sgRNA sequence located next to the proto-spacer adjacent motif, bound by Cas (**a**). The CTG expansion is excised (scissors) via dual CRISPR/Cas9-mediated cleavage, at either side of the repeats. Alternatively (**b**), the transcription of the (CTG)n repeat in DMPK can be prevented by inserting via CRISPR/Cas a premature poly (A) signal between the stop codon and the repeat, leading to premature termination of the transcript (adjusted from [96]). A limitation to this strategy is that the pathogenic repeat remains present in the genome. (**C**). AntagomiR approaches in DM1. MBNL level can be increased by blocking the downregulation of MBNL transcript by miR-23b.

An example of ASO application in DM1 is showed by Wheeler and colleagues, where the morpholino antisense oligonucleotide CAG25 was used to invade CUGexp hairpins to form a stable RNA-morpholino heteroduplex, blockings the formation of CUGexp-MBNL1 complexes (Figure 3A) [116]. A *HSA^LR^* mouse model, with the expanded repeat is in the 3′ UTR of a human skeletal actin (*hACTA1*) transgene [77], was initially used to test the ASO efficacy. Notably, systemic administration of these ASOs directed against the CUGexp RNA caused its rapid knockdown in the skeletal muscle, correcting the physiological, histo-pathological and transcriptomic features of the disease [116]. Reduction of CUGexp RNA was sufficient to release sequestered MBNL1 protein and to improve its splicing regulatory activity toward regulated exons, including *Serca1* exon 22, *Ttn* exon 362, *Zasp* exon 11, and *Clcn1* exon 7a, encoding a chloride ion channel [116]. Remarkably, myotonic release in hindlimb muscles, caused by the repetitive action potentials and delayed muscle relaxation due to the impaired *Clcn1* splicing, were also rescued by the treatment [116]. The effect was sustained for up to 1 year after treatment. Notably, this approach was also successful in a *hDMPK* mouse model, where the ASOs were able to produce significant knockdown of the *hDMPK-CUGexp* transcripts [116]. Interestingly, ASO treatment combined with an exercise training regimen was able to reverse also chronic fatigue, which is the most debilitating symptom of DM1 patients [117]. These promising results support the development of moderate-intensity exercise as an adjuvant for targeted molecular therapies of DM1.

Despite the encouraging results obtained with the ASOs technology in animal models, only one drug entered clinical trials (ClinicalTrials.gov identifier: NCT02312011), but showed limited effects on patients, due to the low concentrations of effective drug reaching the muscle tissue (Myotonic Dystrophy Foundation Annual Conference; www.myotonic.org/digital-academy/ionis-pharmaceuticals, accessed on 17 October 2021, industry-updates-drug-development-2018-mdf-annual-conference).

#### 5.3.2. CRISPR/Cas9 Based Therapies for DM1

Gene editing represents an alternative strategy to correct mutations responsible for inherited disorders, including DM1. The clustered regularly interspaced short palindromic repeats (CRISPR-Cas9) system, initially identified in bacteria as part of the bacterial immune system [118] represents a powerful versatile tool for medical applications [119]. 

In particular, the CRISPR/Cas approach has shown great potential for DM1, as it may ensure a permanent rescue of cell function, solving the disease defect at the DNA level and eliminating downstream toxic effects of the disease. Mechanistically, CRISPR/Cas system interferes with the genome via a small guide RNA (sgRNA), directing the Cas endonuclease to a DNA target that matches the sgRNA sequence located next to the proto-spacer adjacent motif, bound by Cas. Upon binding, Cas protein introduces a double strand break (DSB), normally repaired by the cell through non-homologous end joining (NHEJ). In the case of DM1, the CTG expansion is excised via dual CRISPR/Cas9-mediated cleavage, at either side of the repeat, followed by NHEJ of the two DSBs (Figure 3B) [96,120,121,122]. In addition, the transcription of the (CTG)n repeat in *DMPK* can be prevented by inserting a premature poly(A) signal between the stop codon and the repeat (Figure 3B) [122]. Notably, in this last editing strategy the expanded repeat remains in the genome, possibly favouring heterochromatinization of the *locus* or transcription of an antisense transcript [123]. 

Remarkably, single intramuscular injection of two adeno-associated virus (AAV) vectors expressing the nuclease SaCas9 and selected pair of sgRNAs guide, was also able to delete the CTG repeat in muscle fibers, decreasing the number of myonuclei displaying pathological ribonucleoprotein foci [124]. More recently, Batra and colleagues demonstrated that muscular or systemic injections of AAV vectors encoding nuclease-dead Cas9 and a single-guide RNA targeting CUG repeats results in the expression of the RNA-targeting Cas9 for up to three months, redistribution of MBNL1, elimination of foci of toxic RNA, rescue of splicing defects and amelioration of myotonia [125]. These remarkable results open the path to novel in vivo genome editing strategies for DM1.

#### 5.3.3. AntagomiRs in DM1

One key feature of DM1 is the deprivation of MBNL function due to its sequestration in nuclear foci by CTG expansion. Thus, a potential therapeutic avenue is represented by the increase of MBNL expression, to restore its function. Indeed, MBNL1 overexpression is sufficient to rescue DM1-associated splicing defects and muscle myotonia in the *HSA^LR^* mice [77,126]. Hence, targeting MBNL translational repressors could represent another realistic therapeutic opportunity (Figure 3C). Oligonucleotide-based modulation of miRNA activity by antagomiR has received large attention because of its efficacy in animal models [127]. To this regard, Cerro-Herreros and colleagues firstly identified miR-23b and miR-218 as endogenous translational repressors of MBNL1/2 and MBNL2 [97]. Next, they demonstrated that antagomiR transfection was sufficient to upregulate MBNL proteins and rescue AS in normal and DM1 human myoblasts [97]. Furthermore, antagomiR systemic administration in *HSA^LR^* mice upregulated MBNL proteins in both gastrocnemius and quadriceps muscles and rescued the molecular, cellular, and functional defects of DM1 muscle [97,128]. 

Notably, antagomiR lead candidate (Arthex-01) against miR-23b is on preclinical evaluation by the ARTHEx Biotech, with clinical trials planned by the beginning of 2022 [129].

Therapeutic strategies targeting the alterations associated with NM-disorders are summarized in Table 2.

## 6. Conclusions

AS is a highly regulated and sophisticated process of gene expression that deeply increases proteome diversity. However, it is prone to errors, leading to mis-splicing events, frequently implicated in a wide range of human diseases, including cancer, neurodegenerative diseases, and muscular dystrophies. Understanding the mechanisms underlying splicing defects and the connection to human disease is instrumental for designing effective therapeutic strategies. 

The advent of new technologies, including next generation sequencing (NGS)-based RNA sequencing (RNA-seq), has opened the path to the identification of new causal genes, with a novel focus on RNA mis-splicing. These techniques have revolutionized gene expression studies by enabling researchers to measure relative expression changes across the whole genome. The use of RNA instead of DNA in the analysis yields both the expression profile and mutational status, including the characterization of splice variants, antisense transcription, fusion genes, allele-specific expression, RNA editing and other forms of sequence variation in the transcriptome. Clinical trials using splicing modulatory strategies and RNA targeting have produced impressive results. The potential of ASO therapies to treat the genetic cause of DMD, SMA and other previously untreatable diseases, makes this an exciting opportunity for RNA-based therapeutics and for addressing previously inaccessible drug targets [101]. 

Despite these encouraging results, since the first approval of fomivirsen in 1998 by the FDA for treating cytomegalovirus (CMV) retinitis [130], only 11 ASO-based drugs have received authorization to be used in humans. As mentioned above, limited effects have been achieved in the case of DMD, and the approval of eteplirsen by the FDA was accompanied by controversy due to the high accumulation of dystrophin in the kidneys and rapid urine excretion together with difficulties in quantifying its expression, leaving doubt on its efficacy [131]. Thus, the only ASO-based therapeutic successfully approved for a neurological disease remains nusinersen, used for the treatment of SMA [132]. Nusinersen targets the alternatively spliced exon 7 of *SMN2* pre-mRNA, increasing exon inclusion and producing a functional SMN protein. It is administered directly to the cerebral spinal fluid surrounding the spinal cord by intrathecal injection, with direct uptake into the CNS, allowing circumvention of liver metabolism and kidney excretion [133]. Patients, especially young pre-symptomatic patients, report extended survival over the expected natural history of the disease [133]. 

Hence, several challenges need to be overcome to broaden the clinical use of ASOs, especially in the context of pediatric disorders. In fact, ASOs typically accumulate in organs like kidney, liver, and spleen, resulting in high toxicity, not well tolerated from the young patients.

Moving forward with ASO-based therapies there will be a need to develop non-invasive procedures for an effective delivery of drug and novel chemistries to enhance cellular uptake. These efforts let us hope for a positive outcome on the development of next generation antisense drugs for several genetic diseases.

## Figures and Tables

**Table 1:** Summary of the epidemiological and clinical features of neuromuscular diseases.

Disease	Epidemiology	Age at Onset	Symptoms and Life Expectancy
Congenital myasthenic syndrome (CMS)	Estimated 2.5 to 12 per 1,000,000 individuals	Intrauterine and congenital onset to rarely adolescence	Severe generalized muscle weakness causes bulbar, ocular, limbs and respiratory muscles failure. Heart block leads early death.
Duchenne muscular dystrophy (DMD)	<10 per 100,000 in male <1 per million in female	2 to 6 years	Muscle weakness and wasting affect pelvis, upper arms, and upper legs. Most patients need wheelchair and assisted ventilation before the age of 20. Rarely optimal treatments for cardiopulmonary dysfunction extend life expectancy to late thirties.
Becker muscular dystrophy (BMD)	<8 per 100,000 in male <1 per million in female	Adolescence to early adulthood	Symptoms are almost identical to Duchenne, but less severe and progress slowly. Life quality and expectation is similar to healthy individuals.
Myotonic dystrophy type 1 (DM1)	0.5 to 18 per 100,000 individuals	Birth to adulthood	Progressive weakness, atrophy of distal muscles and myotonia. Affected neonate shows hypotonia (floppy infant syndrome) and need intubation immediately after birth.

**Table 2:** Summary of the therapeutic approaches targeting alterations associated with NM-diseases.

Disease	Gene	Alteration Observed	Tissue Expression	Therapeutic Approach
Congenital myasthenic syndrome (CMS)	*CHRNA1*	Inclusion of exon P3A	NMJ	ASOs covering the 5′ splice site promotes the rescue of P3A skipping [102]
	*CHRNE*	Exclusion of exon 6	NMJ	
	*COLQ*	Exclusion of exon 16	NMJ	
	*RAPSN*	Exclusion of exon 1	NMJ	
Duchenne muscular dystrophy (DMD)	*DMD*	Deletion of exon 50 (leads to an out-of-frame mRNA generating a premature stop codon)	Skeletal muscle	ASOs promoting the skipping of exon 51 are in clinical trials [104]
		Deletion of a single exon (44) or multiple exons (44–45) (induces out-of-frame mutations)		Casimersen increases exon 45-skipping
		Deletion of multiple exons (45–55) (leads to in-frame mutation)		Golodirsen increases the skipping of exon 53 [109]
Myotonic dystrophy type 1 (DM1)	*INSR*	Exclusion of exon 11	Skeletal muscle	ASOs directed against the CUGexp RNA promote release sequestered MBNL1 protein, improving its splicing regulatory activity [116] Adeno-associated virus vectors encoding nuclease-dead Cas9 and a single-guide RNA targeting CUG repeats enhance redistribution of MBNL1 and the rescue of splicing defects [125] AntagomiR 23b upregulates MBNL proteins rescuing the molecular, cellular, and functional defects of DM1 muscle [128]
	*CLCN1*	Inclusion of intron 2 and inclusion of exon 7a	Skeletal muscle	
	*DMD*	Exclusion of exon 78	Skeletal muscle	
	*BIN1*	Exclusion of exon 11	Skeletal muscle	
	*SERCA1*	Exclusion of exon 22	Skeletal muscle	
	*RYR1*	Exclusion of exon 70	Skeletal muscle	
	*CACNA1S*	Exclusion of exon 29	Skeletal muscle	
	*α-DB1*	Inclusion of exon 11A and exon 12	Skeletal muscle	
